# Clinical nutrition in surgical oncology: Young AIOM-AIRO-SICO multidisciplinary national survey on behalf of NutriOnc research group

**DOI:** 10.3389/fnut.2023.1045022

**Published:** 2023-04-14

**Authors:** Luigi Marano, Federica Marmorino, Isacco Desideri, Ludovico Carbone, Alessandro Rizzo, Viola Salvestrini, Franco Roviello, Saverio Cinieri, Vittorio Donato, Raffaele De Luca, NutriOnc Research Group, Silvia Sofia

**Affiliations:** ^1^Department of Medicine, Surgery and Neurosciences, University of Siena, Siena, Italy; ^2^Unit of Oncology, Department of Translational Research and New Technologies in Medicine and Surgery, University of Pisa, Pisa, Italy; ^3^Department of Biomedical, Experimental and Clinical Sciences “Mario Serio”, University of Florence, Florence, Italy; ^4^Don Tonino Bello Oncology, IRCCS Istituto Tumori Giovanni Paolo II Bari, Bari, Italy; ^5^CyberKnife Center, Istituto Fiorentino di Cura ed Assistenza, Florence, Italy; ^6^Medical Oncology Division and Breast Unit, Antonio Perrino Hospital, Brindisi, Italy; ^7^Department of Image Diagnostics, Ospedale San Camillo Forlanini, Rome, Italy; ^8^Department of Surgical Oncology, IRCCS Istituto Tumori “Giovanni Paolo II”, Bari, Italy

**Keywords:** clinical nutrition, cancer, malnutrition, nutritional assessment, survey

## Abstract

Malnutrition is a common condition in cancer patients which is usually associated with functional limitations, as well as increased morbidity and mortality. Based on the support of the young sections of Italian Association of Medical Oncology (AIOM), Italian Association of Radiotherapy and Clinical Oncology (AIRO) and Italian Society of Surgical Oncology (SICO) merged into the NutriOnc Research Group, we performed a multidisciplinary national survey with the aim to define the awareness of nutritional issues among healthcare professionals delivering anticancer care. The questionnaire was organized in four sections, as follows: Knowledge and practices regarding Nutritional Management of cancer patients; Timing of screening and assessment of Nutritional Status; Nutritional Treatment and prescription criteria; Immunonutrition and educational topics. The modules focused on esophagogastric, hepato-bilio-pancreatic and colorectal malignancies. Overall, 215 physicians completed the survey. As regards the management of Nutritional Status of cancer patients, many responders adopted the ERAS program (49.3%), while a consistent number of professionals did not follow a specific validated nutritional care protocol (41.8%), mainly due to lack of educational courses (14.5%) and financial support (15.3%). Nearly all the included institutions had a multidisciplinary team (92%) to finalize the treatment decision-making. Cancer patients routinely underwent nutritional screening according to 57.2% of interviewed physicians. The timing of nutritional assessment was at diagnosis (37.8%), before surgery (25.9%), after surgery (16.7%), before radiochemotherapy (13.5%) and after radiochemotherapy (7%). Most of the responders reported that nutritional status was assessed throughout the duration of cancer treatments (55.6%). An important gap between current delivery and need of nutritional assessment persists. The development of specific and defined care protocols and the adherence to these tools may be the key to improving nutritional support management in clinical practice.

## 1. Introduction

In the era of precision medicine, the "one-size-fits-all" approach has been revolutionarily replaced by a patient-centric vision, which aims at minimizing adverse events while enhancing therapeutic impact of new treatments (1). Despite the current period of innovation in oncology, including big data, genomic sequencing, and minimally invasive surgery as part of standard treatment for some cancers, nutritional issues are still serious clinical challenges (2–7).

It has been estimated that up to 80% of cancer patients suffer from malnutrition (8–12), but less than 30% receive nutritional interventions (13). Anyway, the range of reported data is extremely wide due to the lack of fundamental consensus on malnutrition diagnosis (14–16), as well as differences among several types of solid tumors (17, 18). Therefore, based on a consensus process, the Global Leadership Initiative on Malnutrition (GLIM) has recently suggested a shared method, termed the GLIM-process, for diagnosing malnutrition (19). To this address, patients with digestive tract cancers have been found to have one of the highest rates of malnutrition due to its local effects on bowel function from obstruction and malabsorption, and on glandular secretion from dysfunction and atrophy (20–23). Prior studies suggest that the severity of weight loss is significantly associated with advanced cancer (24), but over 40% of patients are malnourished or at risk for malnutrition, even in early disease stages (11).

Malnutrition is correlated with a prolonged hospital stay, increased morbidity and readmission, as well as higher mortality rates (13, 25–27). Furthermore, malnourished cancer patients may experience a reduced tolerance to chemotherapy, lower quality of life, and reduced overall survival (17, 28–31). As a result, clinical nutrition (32), plays an active role in modern cancer surgery hindering all the adverse effects of cancer-associated malnutrition (33). However, the attitude toward this issue varies considerably among oncologists, surgeons, and radiotherapists due to the lack of structured collaboration between healthcare professionals (34, 35).

Despite the European Society for Clinical Nutrition and Metabolism (ESPEN) recently emphasized individualized plans focused on increasing nutritional intake (31), the European Society of Surgical Oncology (ESSO) and its Young Alumni Club (EYSAC) reported in a global survey that surgical units often lack a structured nutritional assessment, postulating a significant impact on the clinical practice by the involvement of nutritionist medical doctors into the multidisciplinary tumor board (36).

We provide, for the first time ever, an intersociety and multidisciplinary national survey, supported by young sections of *Italian Association of Medical Oncology* (AIOM), *Italian Association of Radiotherapy and Clinical Oncology* (AIRO) and *Italian Society of Surgical Oncology* (SICO) merged into the *NutriOnc Research Group* (Figure 1).

Our survey aims at defining the awareness of nutritional issues in oncological setting among anticancer professionals, mainly focused on esophagogastric (EG) hepato-bilio-pancreatic (HPB) and colorectal (CR) cancers. Of note, in EG and HPB tumors, malnutrition is present in up to 70% of patients, and multimodal anticancer treatments are mandatory, laying the groundwork for a more horizontal and crosscutting shared multimodal nutritional interventions.

FIGURE 1
NutriOnc Research Group. email: nutrionc@gmail.com; LinkedIn: NutriOnc Research Group; Twitter: @NutriOncRG.

## 2. Methods

### 2.1. Survey design

In 2020, young sections of AIOM, SICO and AIRO planned common research goals and founded the NutriOnc Research Group, aimed at implementing multidisciplinary strategies to improve patient quality of life. A web-based survey consisting of a total of 15 multiple choice questions was conducted among young (≤40 years) medical oncologists, surgeons and radiotherapists or young trainees who were AIOM, SICO, AIRO members.

The questionnaire was organized in the following sections: 1. Knowledge and practices regarding Nutritional Management of cancer patients, 2. Timing of screening and assessment of Nutritional Status, 3. Nutritional Treatment and prescription criteria, and 4. Immunonutrition (IMN) and Educational topics.

- Section 1: Use of internal protocols and presence of nutritionists/dieticians as part of the multi-disciplinary team: medical nutritionist/physicians with clinical expertise in nutritional assessment, medical oncologist, surgeon, radiotherapist and trained nurse.
- Section 2: Pre-and In-hospital Nutritional Screening and Assessment, who oversees patients' nutritional assessment, when nutritional assessment is conducted, and which questionnaires are administered.
- Section 3: Post-Hospital Nutritional Management, who oversees activating a home therapeutic plan, what kinds of nutritional therapy are planned, what are the main discomforts encountered by patients.
- Section 4: The level of knowledge of IMN or Immunoenhanced nutrition, suggestions for further discussion.

The modules were mainly focused on EG, HPB, and CR cancers.

### 2.2. Survey dissemination

All young (≤ 40 years) professionals were encouraged to participate by completing modules according to their practice. The questionnaire was distributed online on the AIOM, AIRO and SICO websites in a reserved section and was accessible only through a direct link sent by email on SurveyMonkey®. Overall, the survey could be accessed online from June 30th, 2021, to July 10th, 2021. Responses were anonymously evaluated and analyzed.

### 2.3. Statistical analysis

This survey considered the sample of participants who answered the questionnaire, and therefore no sample size was calculated for a specific hypothesis test. Summary statistical measures for continuous and categorical data were used to describe the sample of doctors' characteristics and their answers to each specific item. Results were compared among different societies. Given the descriptive and exploratory intent of the analysis, no attempt to control for multiple tests was pursued.

## 3. Results

All of the 939 health care professionals representing the target population received the invitation, and 215 (21.5%) participated to the online survey. An overview of participants' demographics and geographic distribution is provided in Table 1.

Among 215 respondents' young health professionals, 101 (47%) were members of SICO, 56 (26%) of AIOM and 45 (21%) of AIRO, while 13 (6%) were not part of any society (Figure 2). The findings regarding the medical specialty were consistent with the results of the membership of SICO, AIOM and AIRO: surgeons (53%) medical oncologist (26.5%), and radiation oncologist (20.4%). Most of the participants were residents (38.1%) and physicians with less and equal to/more than 5 years of medical experience were 67 (31.2%) and 66 (30.7%), respectively.

Concerning the geographical distribution of the responders, 59 were from the Tuscany region, 29 were from Puglia, 20 from Lazio and 17 from Campania (Figure 3). In general, participants were equally distributed between north, center and south-insular Italy.

The physicians dealt with gastrointestinal cancers, and they were focused on CR (61%), EG (24.2%) and HPB (14.8%) cancers. Most of them (34.9%) defined the volume per year of locally advanced CR patients in their own institution as more than 100 patients. Sixty (27.9%) and 48 (22.3%) respondents treated more than 40 HPB and EG l cancer patients per year, respectively. Nearly half of the respondents adopted up-front surgery (42.5%) while 57.5% planned neoadjuvant treatments (radio/chemotherapy).

### 3.1. Knowledge and practices regarding nutritional management of cancer patients (Table 2; Q1-Q4)

Concerning the management of Nutritional Status of cancer patients, many respondents adopted the Enhanced Recovery After Surgery (ERAS) program (49.3%). Of note, a large number of specialists did not follow a specific validated nutritional care protocol (41.8%) due to a lack of educational courses (14.5%), financial support (15.3%) and understaffing (13%). Only 50.7% of health professionals were aware of specific protocols tailored to diagnose and treat malnutrition in cancer patients undergoing neoadjuvant treatment, because there is a practically lack of medical

TABLE 1 Participants' demographics and institutional volumes [*n* (%)].

| | *n* (%) |
|---|---|
| Societies | |
| SICO | 101 (47) |
| AIOM | 56 (26) |
| AIRO | 45 (21) |
| Other | 13 (6) |
| Role of participants | |
| Residents | 82 (38.14) |
| Consultant 0–5 years | 67 (31.16) |
| Consultant 5–10 years | 66 (30.7) |
| Geographic distribution | |
| *Northwest Italy* | 34 |
| Liguria | 0 |
| Lombardy | 22 (10.23) |
| Piedmont | 12 (5.58) |
| Val d'Aosta | 0 |
| *Northeast Italy* | 28 |
| Emilia Romagna | 11 (5.12) |
| Friuli Venezia Giulia | 3 (1.4) |
| Trentino Alto Adige | 2 (0.93) |
| Veneto | 12 (5.58) |
| *Central Italy* | 82 |
| Marche | 3 (1.4) |
| Lazio | 20 (9.3) |
| Tuscany | 59 (27.44) |
| Umbria | 0 |
| *South Italy* | 58 |
| Abruzzo | 4 (1.86) |
| Basilicata | 1 (0.47) |
| Calabria | 5 (2.23) |
| Campania | 17 (7.91) |
| Molise | 2 (0.93) |
| Puglia | 29 (13.49) |
| *Insular Italy* | 13 |
| Sardinia | 6 (2.79) |
| Sicily | 7 (3.28) |
| Activities of the unit | |
| Gastroesophageal | 52 (24.19) |
| Colorectal | 131 (60.93) |
| Hepato-bilio-pancreatic | 32 (14.88) |
| Institutional volumes | |
| > 100 patients with localized or locally advanced disease treated per year | 75 (34.88) |
| Gastroesophageal cancer | 162 (75.35) |
| > 40 | 60 (27.91) |
| 20–40 | 42 (19.53) |
| < 20 | 60 (27.91) |
| Colorectal cancer | 189 (87.91) |
| > 100 | 75 (34.88) |
| 60–100 | 64 (29.77) |
| < 60 | 50 (23.26) |
| Hepato-bilio-pancreatic cancer | 139 (64.66) |
| > 40 | 48 (22.33) |
| 20–40 | 45 (20.93) |
| < 20 | 46 (21.40) |

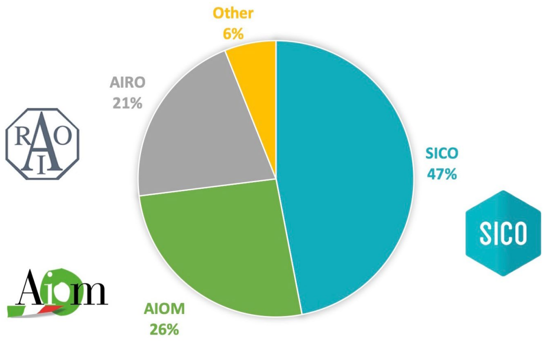

FIGURE 2
Affiliations of participants. Italian Association of Medical Oncology (AIOM), Italian Association of Radiotherapy and Clinical Oncology (AIRO) and Italian Society of Surgical Oncology (SICO).

nutritional specialist in the institutions. Of note, nearly all the Institutions had a multidisciplinary team (92%) to finalize the treatment decision-making. In the absence of dietician, the nutritional assessment of patients performing oncological care was managed by physicians with clinical expertise in nutritional (76.4%).

### 3.2. Timing of screening and assessment of nutritional status (Table 3; Q5-Q8)

Cancer patients underwent nutritional screening for 57.2% of respondents. In the absence of nutritional staff as described before, the nutritional screening was managed by physicians with clinical expertise in nutritional assessment (35.6%), by surgeons (34.3%), medical/radiation oncologists (36.8%) and by case-manager nurses (6.8%) (Figure 4A). The timing of nutritional assessment was at the first visit (37.8%), before surgery (25.9%), after surgery (16.7%), before radiochemotherapy (RT-CT) (13.5%) and after RT-CT (7%). Most of the respondents reported that nutritional status was assessed throughout the duration of cancer treatments (55.6%) (Figure 4B). Knowledge of nutritional screening tools was reported by 63.3% of physicians. Malnutrition Universal Screening Tool (MUST), Malnutrition Screening Tool (MST), Nutritional Risk Screening 2002 (NRS – 2002), Mini Nutritional Assessment Short-Form (MNA-SF) and Global Leadership Initiative on Malnutrition (GLIM) criteria were known by 56.1, 15, 30.8 and 15.7% of cases. Moreover, 23 (15.7%) respondents were aware of body composition analysis by means of bioimpedance analysis (Bioelectrical Impedance Vector Analysis, BIVA) and 24 (16.4%) of computed tomography (CT) scan of L3 vertebral body as nutritional screening tools (Figure 4C). Nutritional assessment has been performed in the 81.8% of cases reporting abnormal results at nutritional screening. The physicians reported the periodical assessment of the nutritional status of cancer patients during oncological care (60.5%). Concerning the timing, 31.3% conducted the nutritional assessment at diagnosis, 28.1% before surgery, 14.5% after surgery, 20.5% before RT-CT and 8.1% after RT-CT (Figure 4D). Haematochemical indices, such as serum albumin, pre-albumin, creatinine, iron levels, 3-methylhistidine, cholesterol and total lymphocyte (37) (54.4%), food diary (59.3%) and MNA-SF (38.9%) were the main methods adopted by the responders (Figure 4E).

### 3.3. Nutritional treatment and prescription criteria (Table 4; Q9-Q11)

After discharge from the hospital, the prescription of nutritional support was provided by dieticians (59.5%), medical oncologists (20%), surgeons (15.3%), radiation oncologists (5.12%) (Figure 4F). The respondents were asked about the main issues regarding nutritional support facilities, available in their institutions. Most of them (44.2%) identified the difficulty of finding nutritional products in hospital pharmacies and for patients to have refundability as the main crucial factors. Seventy-seven (35.8%) specialists declared fatigue and loss of appetite as crucial factors concerning nutritional therapy. Similarly, respondents mentioned that nausea (29.3%), malabsorption (24.1%), dysphagia (21.8%), vomiting (18.6%) and mucositis (11.1%) negatively affected the management of nutritional therapy. Additionally, the absence of a dietician/nutritionist in the department (28.3%) as well as the lack of specific rules concerning the prescription criteria of nutritional support (13%) were described as further key issues as factor negatively affecting the optimal nutritional management. In cases of malnutrition (e.g., more than 5% of weight loss in the last 3 months) (38), the physician choices were nutritional counseling (34.8%), oral nutritional supplement (28.8%), enteral nutrition (2.8%), parenteral nutrition (3.3%), and no treatments (0.9%).

### 3.4. Immunonutrition and educational topics (Table 5; Q12-Q15)

During radiotherapy, 95% of physicians declared that patients needed a supplementary nutrition. Arginine, Omega-3 fatty acids (omega-3 Fas), vitamins/antioxidants, Glutamine, Whey Protein (WP) and RNA were described as essential supplements during radiotherapy by 29.7, 39.5, 49.8, 23.3, 20.9 and 4.7% of respondents, respectively. Fifty-eight (27%) physicians stated that supplementation was not necessary during radiotherapy. Knowledge of IMN was reported by 58.1% of respondents. IMN was prescribed perioperatively (54.5%), before surgery (36.3%) and during neoadjuvant RT-CT (22.3%). One hundred physicians expressed interest in educational courses addressing nutritional issues as a whole. Webinar were mentioned as preferred modality for educational programs (78.1%).

## 4. Discussion

According to our results based on 215 physicians, almost half of the respondents declared to adopt the ERAS (Enhanced Recovery After Surgery) program (49.3%), while an important number of professionals did not follow a specific validated nutritional care protocol (41.8%) due several reasons, such as lack of educational

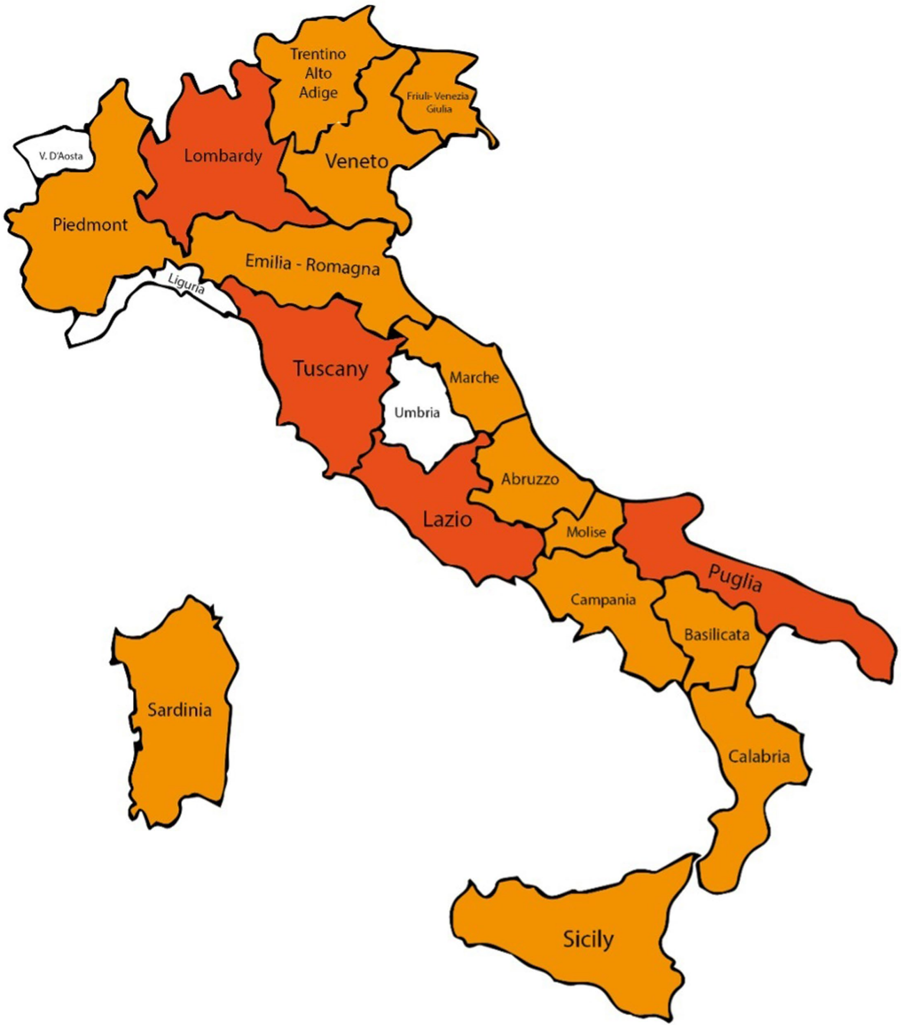

FIGURE 3
Italian map of survey participants: regions with >20 interviewees in dark orange, with 1–19 interviewees in orange, otherwise in white.

courses (14.5%) and financial support (15.3%). In addition, nearly all the included centers had a multidisciplinary team (92%) and the timing of nutritional assessment was at diagnosis (37.8%), before surgery (25.9%), after surgery (16.7%), before radiochemotherapy (RT-CT) (13.5%) and after RT-CT (7%). Moreover, nutritional status was assessed throughout the duration of cancer treatments in 55.6% of cases.

Cancer-related malnutrition is very common among patients affected by solid malignancies, with a high percentage of patients experiencing malnutrition during the course of their disease due to several reasons, including the type of tumor, the disease stage and the type of treatment (e.g., surgery, chemotherapy, concomitant RT-CT, etc.) (39–41). Thus, it is mandatory to identify cancer patients at nutritional risk as a component of a proper, comprehensive care (42). Based on these premises, the delivering of optimal nutritional care is a crucial need for cancer patients. Early detection of nutritional status is essential to initiate nutritional treatment for preserving body composition and avoiding sarcopenia and cachexia, as the toxicity of anticancer therapies depends on body cell mass, and oncological clinical outcomes could be negatively related to malnutrition regardless of therapies (43–46).

In the current survey, we aimed to define the awareness of nutritional issues among young healthcare professionals delivering anticancer care, by providing an intersociety and multidisciplinary national survey supported by young sections of different associations. Despite a notable participation, nutritional screening was carried out at diagnosis by less than 40% of anticancer professionals. This is considered the first step in defining patients at risk of malnutrition or malnourished, followed by a nutritional assessment that may more deeply investigate and schedule the patients to a tailored nutritional support. As previously reported, only 50.7% of responders were aware of specific tools to diagnose timely malnutrition in cancer patients undergoing neoadjuvant treatment. An increasing awareness of nutritional pathway, especially in the surgical community (64.3%), is related to the wide

10.3389/fnut.2023.1045022

TABLE 2 Section 1: Knowledge and practices regarding nutritional management of cancer patients. Questionnaire answer distribution [*n* (%)].

| | All (*n*=215) | SICO (*n*=101) | AIOM (*n*=56) | AIRO (*n*=45) |
|---|---|---|---|---|
| Section 1. Knowledge and practices regarding Nutritional Management of cancer patients | | | | |
| Q1. Does the centre where you work already have a specific internal protocol for the clinical nutrition of patients? * | | | | |
| Yes (ERAS) | 106 (49.3%) | 65 (64.36%) | 20 (35.71%) | 16 (35.56%) |
| Yes (others) | 22 (10.23%) | 6 (5.94%) | 9 (16.07%) | 7 (15.56%) |
| NO | 90 (41.86%) | 31 (30.69%) | 27 (48.21%) | 24 (53.33%) |
| Q2. Are there nutritional protocols for the malnourished cancer patient who undergoes neoadjuvant therapy? | | | | |
| Yes | 101(46.97%) | 42 (41.59%) | 21 (37.5%) | 30 (68.67%) |
| No | 77 (35.81%) | 41 (40.59%) | 20 (35.71%) | 12(26.67%) |
| Only in case of Gastro-Esophageal tumors | 23 (10.7%) | 9 (8.91%) | 11 (19.64%) | 2 (4.44%) |
| Only in case of Colorectal tumors | 7 (3.26%) | 5 (4.95%) | 1 (1.79%) | 1 (2.22%) |
| Only in case of Hepato-bilio-pancreatic tumors | 4 (1.86%) | 2 (1.98%) | 2 (3.57%) | 0 |
| Others | 3 (1.4%) | 2 (1.98%) | 1 (1.79%) | 0 |
| Q3. Is there a multidisciplinary team in your centre that evaluates cancer patients? | | | | |
| Yes | 198 (92.09%) | 89 (88.12%) | 52 (92.86%) | 45(100%) |
| No | 17 (7.91%) | 12 (11.88%) | 4 (7.14%) | 0 |
| Q4. If there is no nutritionist: do you have a consultant? | | | | |
| Yes | 133 (76.44%) | 61 (74.39%) | 39 (81.25%) | 27 (77.14%) |
| No | 41 (23.56%) | 21 (25.61%) | 9 (18.75%) | 8 (22.86%) |

*More than one answer was eligible.

adoption of ERAS protocol (47). Interestingly, no ERAS guidelines consider a specific nutritional protocol for patients scheduled to neoadjuvant treatment, but only perioperatively. Interestingly, this timeframe could represent a "window of opportunity" to define a specific and tailored protocol, to improve treatment compliance, quality of life, and to complete the specific neoadjuvant treatment. Fewer than 60% of the surveyed professionals report that nutritional assessment was carried out continuously throughout the therapeutic care pathway, which calls for its implementation and increased use. The current survey underlines the importance of proper nutritional screening and assessment by health professionals and highlights that an important gap between current delivery and the need for nutritional assessment and care persists. Some strengths and limitations of the current survey should be highlighted. Among the strengths of this study, it is worth noting that this survey included the young sections of three different anticancer Italian associations (AIOM, AIRO, and SICO, merged into the NutriOnc Research Group) and an overall high number of included professionals and Institutions while its limitations include the lack of data on clinical outcomes. Our findings are partially in line with previous experiences suggesting that a remarkable proportion of interviewed professionals does not follow a specific nutritional protocol in the daily clinical practice due to several reasons (e.g., lack of financial support, lack of educational courses, lack of human resources) (48). The value of using different screening tools has practical limitations: parameters should be easy, quick, useful in different populations, and carried out by both dieticians and physicians with clinical expertise in nutritional assessment. Our group would encourage the use of single, shared screening tools, such as MST, to screen all adults for malnutrition in all settings, according to the last position of the Academy of Nutrition and Dietetic (39).

As previously reported, nutritional screening has been performed globally in the 57.2%, in low percentage by a nutritionist, while in high percentage by surgeons, oncologists and radiotherapists or physicians with "an experience in nutritional issue." The nutritional assessment was performed by nurse case managers in only 6.8% of cases. On the one hand, these data suggest the poor inclusion of nutritionist medical specialists in several centers, while these figures should be considered of pivotal importance in this setting. On the other hand, given the lack of financial support and human resources, the disappointing data regarding nurse case managers poses some reflections. In our opinion, this health care figure should be more actively engaged in the nutritional pathway of cancer patients, ranging from the diagnosis to the follow-up of cancer. The central role of nurses has been also suggested by a study published by McHugh and colleagues, since the presence of specialized nurses has an impact in terms of better outcomes, reduced length of hospital stay, less readmissions, and even a decreased mortality rate (49).

At the same time, the current study underlines a higher awareness of nutritional assessment as a fundamental component of cancer patients care in younger health professionals compared with previous multicenter reports, including the study published by Caccialanza and colleagues (34). In this previous Italian experience, the authors observed that nutritional assessment was performed by less than 30% of oncologists at diagnosis, a percentage which is significantly lower compared to that observed in the current survey. According to 42% of oncologists included in this previous Italian study, nutritional assessment was carried out only after patients requested it, and almost 60% of patient affiliates were not aware of

10.3389/fnut.2023.1045022

TABLE 3 Section 2: Timing of screening and assessment of Nutritional Status. Questionnaire answer distribution [*n* (%)].

| | All (*n*=215) | SICO (*n*=101) | AIOM (*n*=56) | AIRO (*n*=45) |
|---|---|---|---|---|
| Section 2. Timing of screening and assessment of Nutritional Status | | | | |
| Q5. Does your center carry out nutritional screening of patients? | | | | |
| Yes | 123 (57.21%) | 53 (52.48%) | 30 (53.57%) | 33 (73.33%) |
| No | 92 (42.79%) | 48 (47.52%) | 26 (46.43%) | 12 (26.67%) |
| Q6. Who is responsible for the nutritional screening of patients if there is no nutritionist/dietician? | | | | |
| A physician with experience in nutritional assessment | 57 (35.63%) | 21 (28.77%) | 19 (39.58%) | 13 (40.63%) |
| Nurse Case Manager | 11 (6.88%) | 7 (9.59%) | 2 (4.17%) | 2 (6.25%) |
| The surgical team | 55 (34.38%) | 47 (64.38%) | 3 (6.25%) | 3 (9.38%) |
| Medical Oncologist/Oncologist Radiotherapist | 59 (36.88%) | 10 (13.70%) | 29 (60.42%) | 19 (59.38%) |
| Q7. Do you know any nutritional screening method? * | | | | |
| Yes | 136 (63.26%) | 67 (66.33%) | 35 (62.5%) | 26 (57.78%) |
| No | 79 (36.74%) | 34 (33.67%) | 21 (37.5%) | 19 (42.22%) |
| Yes (Malnutrition Universal Screening Tool, MUST) | 82 (56.16%) | 39 (54.93%) | 24 (61.54%) | 16 (55.17%) |
| Yes (Malnutrition Screening Tool, MST) | 22 (15.07%) | 11(15.49%) | 9 (23.08%) | 1 (3.45%) |
| Yes (Nutritional Risk Screening 2002, NRS-2002) | 36 (24.66%) | 28 (39.44%) | 3 (7.69%) | 5 (17.24%) |
| Yes (Mini Nutritional Assessment Short-Form, MNA-SF) | 45 (30.82%) | 19 (26.76%) | 15 (38.46%) | 8 (27.59%) |
| Yes (GLIM criteria) | 23 (15.75%) | 11 (15.49%) | 5 (12.82%) | 7 (24.14%) |
| Yes (instruments: Bioelectrical impedance vector analysis, BIVA) | 23 (15.75%) | 15 (21.13%) | 7 (17.95%) | 1 (3.45%) |
| Yes (instruments: CT at L3 level) | 24 (16.44%) | 17 (23.94%) | 4 (10.26%) | 2 (6.90%) |
| Yes (instruments) | 14 (9.59%) | 6 (8.45%) | 2 (5.13%) | 6 (20.69%) |
| Q8. After having performed the nutritional screening with an abnormal result, is the nutritional assessment carried out? * | | | | |
| Yes | 176 (81.86%) | 76 (75.25%) | 48 (85.71%) | 44 (97.78) |
| No | 39 (18.14%) | 25 (24.75%) | 8 (14.29%) | 1 (2.22%) |
| Yes (Mini Nutritional Assessment Short-Form, MNA-SF) | 65 (38.92%) | 27 (37.50%) | 22 (45.83%) | 13 (32.5%) |
| Yes (Subject Global Assessments, SGA) | 21 (12.57%) | 8 (11.11%) | 7 (14.28%) | 5 (12.5%) |
| Yes (Score patient-generated Subject Global Assessment, PG-SGA) | 24 (14.37%) | 6 (8.33%) | 5 (10.42%) | 11 (27.5%) |
| Yes (GLIM criteria) | 21 (12.57%) | 8 (11.11%) | 5 (10.42%) | 7 (17.5%) |
| Yes (instruments: Bioelectrical impedance vector analysis, BIVA) | 17 (10.18%) | 9 (12.50%) | 6 (12.5%) | 2 (5%) |
| Yes (instruments: CT at L3 level) | 8 (4.79%) | 8 (11.11%) | 0 | 0 |
| Yes (instruments: Blood tests/PCR) | 91 (54.49%) | 47 (65.28%) | 24 (50%) | 17 (42.5%) |
| Yes (instruments) | 18 (10.78%) | 8 (11.11%) | 3 (6.25%) | 6 (15%) |
| Yes (Food diary) | 99 (59.28%) | 42 (58.33%) | 24 (50%) | 28 (70%) |

*More than one answer was eligible.

clinical referrals for home artificial nutrition management. At the same time, for almost all respondents, the nutritional status assessment was considered fundamental in predicting tolerance to anticancer treatment. In another German survey across certified oncologic care institutions, the authors reported that only around 30% of patients received nutritional counseling, regardless of their nutritional status and disease stage, and none of the included centers presented a systematic screening procedure and had paucity of dieticians available (50).

Another point to highlight includes the presence of a multidisciplinary team, which was reported in 92% of the Institutions included in the current survey. This is particularly important since measuring and monitoring metabolic and nutritional status should be reviewed as a defined, standardized part of each multidisciplinary team process, and the presence of specialist oncology dieticians should be encouraged as an essential component of team and involved in all phases of cancer patient management (51, 52). In fact, the high frequency of nutritional issues in each phase of cancer care, ranging

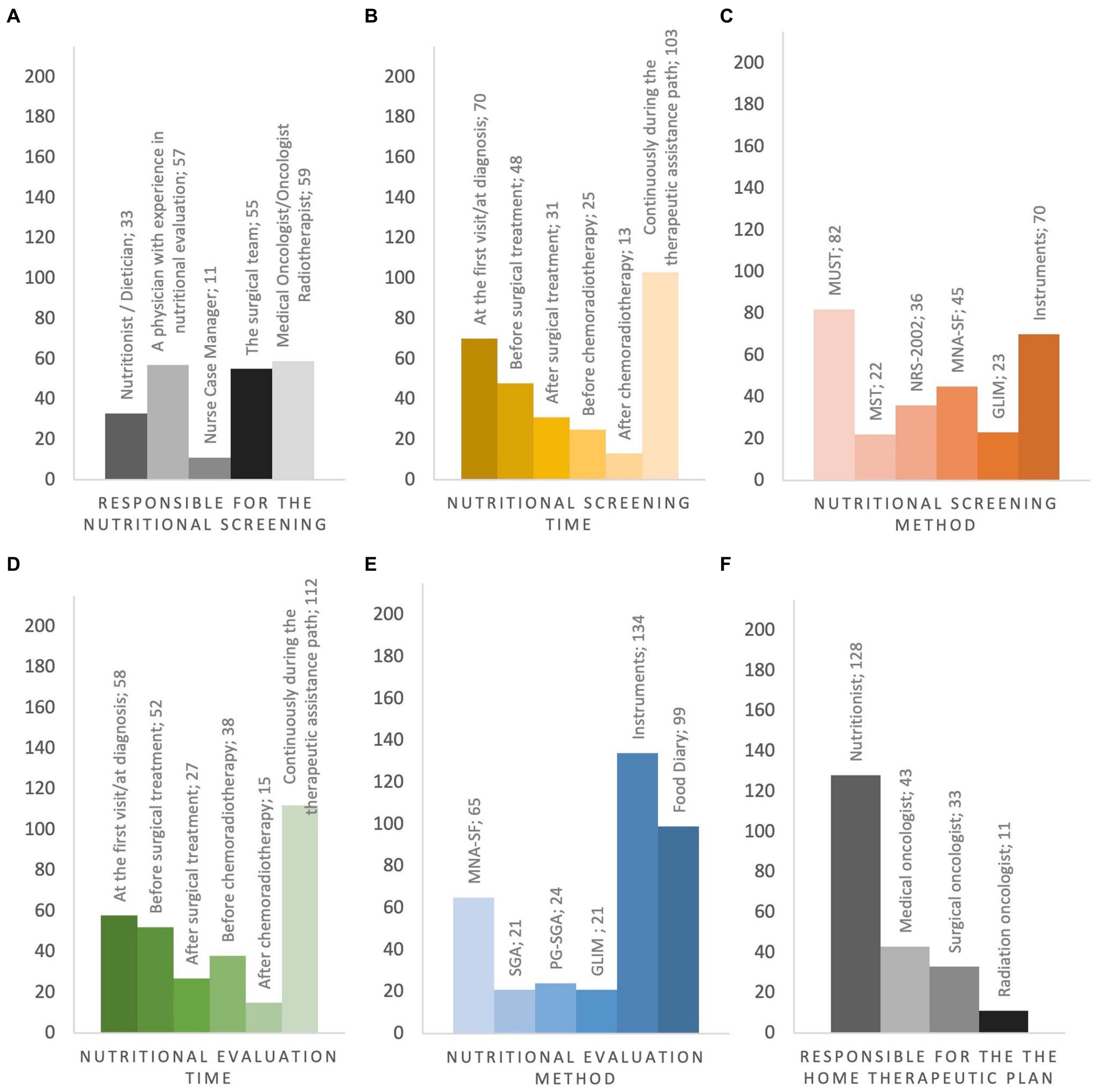

FIGURE 4
Pre-and In-hospital Nutritional Screening and Assessment, Post-Hospital Nutritional Management. **(A)** responsible for the nutritional screening; **(B)** when nutritional screening is performed; **(C)** type of questionnaire administered in nutritional screening; **(D)** when the severity of malnutrition is investigated; **(E)** type of questionnaire administered in nutritional grading assessment; **(F)** who is in charge of activating the home therapeutic plan. MUST Malnutrition Universal Screening Tool, MST Malnutrition Screening Tool, NRS-2002 Nutritional Risk Screening 2002, MNA-SF Mini Nutritional Assessment Short-Form, GLIM Global Leadership Initiative on Malnutrition, SGA Subject Global Assessments, PG-SGA Score patient-generated Subject Global Assessment, Instruments Bioelectrical impedance vector analysis, CT-scan at L3 level, Blood test.

from diagnosis to end of life, makes the integration of nutrition assessment within multidisciplinary teams a key topic.

At the same time, if malnutrition seems to be perceived as a severely limiting factor by both patients and anticancer professionals, adherence to recommendations and guidelines appears low. The development of specific and defined care protocols and the adherence to these shared tools may be one of the keys to improve nutritional support management. Increased awareness of the impact of malnutrition on clinical outcomes is crucial to allow for high-quality nutrition treatments before, during, and following anticancer therapies. In addition, the most commonly used tools are based on the items established in the ERAS protocol being efficient and with adequate specificity and sensitivity, something that should help a wider diffusion of nutritional assessment (25). Of note, through the application of ERAS items protocol IMN has gained more awareness, especially in the perioperative period. However, our survey demonstrated a wide variation among surgeons, oncologist, and radiotherapist in "familiarity" about IMN, while most of the responders consider IMN in a future and common area of interest represented by the impact on clinical outcome in patients undergoing neoadjuvant and surgical treatment and finally cost-effectiveness.

TABLE 4 Section 3: Nutritional Treatment and prescription criteria. Questionnaire answer distribution [*n* (%)].

| | All (*n*=215) | SICO (*n*=101) | AIOM (*n*=56) | AIRO (*n*=45) |
|---|---|---|---|---|
| Section 3. Nutritional Treatment and prescription criteria | | | | |
| Q9. If a nutritional support to be administered at patient's home is necessary who is in charge of activating the therapeutic plan? | | | | |
| Medical oncologist | 43 (20%) | 19 (18.81%) | 20 (35.71%) | 2 (4.44%) |
| Surgical oncologist | 33 (15.35%) | 25 (24.75%) | 1 (1.79%) | 2 (4.44%) |
| Radiation oncologist | 11 (5.12%) | 0 | 0 | 11 (24.44%) |
| Nutritionist | 128 (59.53%) | 57 (56.44%) | 35 (62.5%) | 30 (66.67%) |
| Q10. In the case of a malnourished patient (e.g., with weight loss>5% in less than 3 months) which kind of nutritional therapy is usually given? | | | | |
| Nutritional Counseling | 75 (34.88%) | 23 (22.77%) | 31 (55.36%) | 18 (40%) |
| Integration *per os* | 62 (28.84%) | 34 (33.66%) | 13 (23.21%) | 9 (20%) |
| Enteral *via* PEG | 6 (2.79%) | 3 (2.97%) | 0 | 2 (4.44%) |
| Associated/mixed (os/parenteral) | 63 (29.3%) | 33 (32.67%) | 12 (21.43%) | 15 (33.33%) |
| Partial/total parenteral | 7 (3.26%) | 6 (5.94%) | 0 | 1 (2.22%) |
| Other | 2 (0.93%) | 2 (1.98%) | 0 | 0 |
| Q11. What are the problems you face during the delivery of nutritional therapy? * | | | | |
| Supply and/or Refundability of the product | 95 (44.19%) | 41 (40.59%) | 28 (50%) | 18 (40%) |
| Absence of a therapeutic plan in the regional Risk Assessment Document | 28 (13.02%) | 15 (14.85%) | 10 (17.86%) | 2 (4.44%) |
| Absence of a nutritionist in the ward | 61 (28.37%) | 33 (32.67%) | 18 (32.14%) | 6 (13.33%) |
| Mucositis | 24 (11.16%) | 4 (3.96%) | 8 (14.29%) | 11 (24.44%) |
| Asthenia/loss of appetite | 77 (35.81%) | 31 (30.69%) | 20 (35.71%) | 21 (46.67%) |
| Dysphagia | 47 (21.86%) | 14 (13.86) | 11 (19.64%) | 20 (44.44%) |
| Nausea | 63 (29.3%) | 30 (29.7%) | 10 (17.86%) | 20 (44.44%) |
| Vomiting | 40 (18.6%) | 19 (18.81%) | 8 (14.29%) | 12 (26.67%) |
| Malabsorption | 52 (24.19%) | 28 (27.72%) | 11 (19.64%) | 12 (26.67%) |
| Other | 15 (6.98%) | 9 (8.91%) | 4 (7.14%) | 2 (4.44%) |

*More than one answer was eligible.

As previously stated, if the impact of early nutritional intervention on quality of life and clinical outcomes of cancer patients is widely recognized, some steps forward remain to be taken, and unfortunately, nutritional assessment is frequently neglected. Certainly, healthcare professionals delivering anticancer care have a long way to go to make nutrition care a standardized component of cancer management, but positive signals emanating from this intersociety and multidisciplinary national survey should encourage anticancer professionals to improve systematic nutritional screening and management of oncological patients.

### 5. Conclusion

To the best of the Authors' knowledge, the current study represents the first survey in literature supported by young sections of three healthcare professionals delivering anticancer care organizations from Italy (AIOM, AIRO, and SICO). Although the results could be affected by a not wide response rate from the target population receiving the invitation, our findings could suggest the persistence of an important gap between current delivery and need of nutritional assessment due to several reasons, and nutritional screening was performed globally in the 57.2% of cases, and in low percentage by a nutritionist. Despite the importance of the assessment of nutritional status in cancer patients is widely recognized by anticancer professionals, some questions remain unanswered, and our results call for an implementation, development, and larger use of specific tools able to perform nutritional assessment in this setting, and to improve nutritional support management in clinical practice.

### Data availability statement

The raw data supporting the conclusions of this article will be made available by the authors, without undue reservation.

### Author contributions

All authors listed have made a substantial, direct, and intellectual contribution to the work and approved it for publication.

TABLE 5 Section 4: Immunonutrition and Educational topics. Questionnaire answer distribution [*n* (%)].

| | All (*n*=215) | SICO (*n*=101) | AIOM (*n*=56) | AIRO (*n*=45) |
|---|---|---|---|---|
| Section 4. Immunonutrition and Educational topics | | | | |
| Q12. Do you think there are essential substrates for the nutritional support of patients treated with radiotherapy? | | | | |
| Yes | 95 (44.19%) | 40 (39.60%) | 25 (44.64%) | 21 (46.67%) |
| No | 58 (26.98%) | 34 (33.66%) | 16 (28.57%) | 8 (17.78%) |
| Q13. Are you familiar with immunonutrition? If YES, can you indicate when it is prescribed? * | | | | |
| Yes (Perioperative) | 72 (54.55%) | 54 (60.67%) | 10 (41.67%) | 3 (27.27%) |
| Yes (Preoperative) | 48 (36.36%) | 39 (43.82%) | 4 (16.67%) | 2 (18.18%) |
| Yes (in Radio/Chemotherapy) | 30 (22.73%) | 9 (10.11%) | 12 (50%) | 8 (72.73%) |
| No | 91 (42.33%) | 10 (9.9%) | 36 (64.29%) | 39 (86.67%) |
| Q14. Are there any topics related to nutrition in oncology that you would like to investigate? * | | | | |
| Clinical impact of immunonutrition | 139 (64.65%) | 54 (53.47%) | 42 (75%) | 34 (75.56%) |
| Results of immunonutrition in patients undergoing neoadjuvant treatment | 138 (64.19%) | 56 (55.45%) | 36 (64.29%) | 36 (80%) |
| Surgical outcomes in immunotreated patients | 135 (62.79%) | 81 (80.2%) | 26 (46.43%) | 20 (44.44%) |
| Cost-effectiveness analysis of immunonutrition | 93 (43.26%) | 48 (47.52%) | 23 (41.07%) | 17 (37.78%) |
| Q15. How do you wish to receive information (indicate the methods you prefer)? * | | | | |
| Specific webinar | 168 (78.14%) | 84 (83.17%) | 46 (81.14%) | 28 (62.22%) |
| Clinical work presentation | 78 (36.28%) | 42 (41.58%) | 14 (25%) | 15 (33.33%) |
| Podcast | 30 (13.95%) | 15 (14.85%) | 6 (10.71%) | 7 (15.56%) |
| Infographic card | 40 (18.60%) | 20 (19.8%) | 7 (12.5%) | 7 (15.56%) |
| Information slide set | 79 (36.74%) | 28 (27.72%) | 30 (53.57%) | 17 (37.78%) |

*More than one answer was eligible.

### Group members of NutriOnc Research Group

NutriOnc Research Group Collaborators:

SICO: Silvia Sofia, Marco Milone, Benedetto Ielpo, Maria Teresa Mita, Silvia Ministrini, Mario Giuffrida, Roberta Tutino, Caterina Baldi, Giampaolo Perri, Anna Stella Lippolis, Chiara Marafante, Giusy Giannandrea, Marco Vito Marino, Letizia Laface, Salomone Di Saverio, Luca Aldrighetti, Nicola de'Angelis, Nick Salimian, Marco Caricato, Gianluca Pellino, Sara Vertaldi, Federica Cipriani, Gabriella Teresa Capolupo, Antonio Costanzo, Letizia Santandrea, Gaetano Gallo, Andrea Belli, Laura Mastrangelo, Fausto Rosa, Nicolò Pecorelli, Graziella Marino, Alessio Giordano, Nicola Cillara, Maria Lemma, Francesco Pata, Federico Cammillini, Gianmario Edoardo Poto, Giulia Grassi, Donato Francesco Altomare, Arcangelo Picciariello, Lorenzo Petagna, Luca Ippolito, Elio Treppiedi, Daniele Delogu, Abdallah Moukachar, Stefano Granieri, Giuseppe Cuticone, Osvaldo Carpineto Samorani, Daniela Rega, Leonardo Solaini, Stefano de Pascale, Francesca Ascari, Michele Manigrasso, Simona Badalucco, Salvatore Paiella, Sara Coppola, Roberta Iadarola, Giovanna Di Meo, Isacco Montroni, Fabio Vistoli, Valentina Ferraro, Edoardo Saladino, Federico Fazio, Roberta Rota, Francesco Orlando, Simone Famularo, Cinzia Bizzoca, Giorgio Dalmonte, Marco Inama, Luigi Verre, Leandro Siragusa, Casoni Pattacini Gianmaria, Michele Benedetti, Nicolò Tamini, Cristian Conti, Giorgio Ammerata, Serena Mantova, Vito Leonardo Pinto, Arianna Corvasce, Giorgio Micheletti, Teresa Perra, Marco Pellicciaro, Marco Materazzo, Michele Zuolo, Emanuele Doria, Antonio Brillantino, Luca Del Prete, Andrea Muratore, Claudio Luciani, Giulia Turri, Fabio Casciani, Giuliani Giuseppe, Graziana Barile, Gaia Oldrà, Valeria Restaino, Simona Deidda, Michele Ammendola, Andrea Fares Bucci, Patrizia Marsanic, Dario Cassetti, Luca Resca, Daniele Fusario, Eleonora Andreucci.

AIOM: Anna Michelotti, Brunella Amoruso, Isabella Franco, Laura Noto, Andrea Spallanzani, Raimondo Calogero Scalia, Teresa Del Giudice, Valeria Merz, Gianmarco Motta, Alessandro Parisi, Mikol Modesti, Antonella Argentiero, Debora Basile, Gianmarco Vannini, Carlotta Ottanelli, Salvatore Corallo, Eufemia Stefania Lutrino, Daniele Rossini, Federica Morano, Luigia Stefania Stucci, Costanza Winchler, Martina Catalano, Andrea Marini, Giuseppe Brisinda, Enrico Sammarco, Martina Carullo, Giandomenico Roviello, Mirko Barone, Maria Grazia Rodriquenz, Giuseppe Tirino, Alessia Amoruso, Anna Russo, Veronica Conca, Laura Orgiano, Sveva Macrini, Giulia Nazzicone, Maria Bensi, Martina Montesano, Emanuela Dell'Aquila, Andrea Sbrana, Beatrice Borelli, Lorenzo Fornaro, Lucrezia Raimondi, Valeria Zurlo, Mattia Garutti, Elena Ongaro, Arianna Pellegrino, Andrea lanese, Laura Bernardini, Alessandra Boccaccino, Patrizia Farina, Federica Buzzacchino, Angelica Petrillo, Ada Taravella, Vittorio Studiale, Paolo Ciracì.

AIRO: Giovanna Lovino, Dora Di Cosmo, Sabrina Montrone, Fabiana Gregucci, Luca Dominici, Alba Fiorentino, Filippo Carannante, Giambattista Siepe, Giampaolo Montesi, Manuele Roghi, Michele Aquilano, Andrea Romei, Ilaria Bonaparte, Roberta Grassi, Emma D'Ippolito, Giulio Frosini, Giuseppina De Marco, Gennaro Giovine, Chiara Mattioli, Ilaria Morelli, Victoria Lorenzetti, Matteo Mariotti, Carolina Orsatti, Vincenzo Troncone, Lorenzo Livi, Antonio Angrisani, Marco Banini, Teresa Di Pietro, Giuseppe Carlo Iorio, Iacopo Cavallo, Cecilia Cerbai, Valerio Nardone, Francesca De Felice, Consuelo Rosa, Giulia Stocchi, Sara Lucidi, Michele Ganovelli, Damiano Dei, Chiara Cascone, Anna Peru, Luisa Caprara, Lucia Angelini, Luca Visani, Giulio Francolini, Beatrice Bettazzi.

Non-affiliated: Francesco Belia, Virginia Boccardi, Simone Serafini, Vincenzo Bottino, Luca Ferrario, Giuseppe Frazzetta, Felice Pirozzi, Gennaro Martines, Francesco Antonio Ciarleglio, Daniele Indiani, Giuliano Barugola, Luca Mazza, Chiara Guarini.

### Funding

The study was received funding from Nestlè Health Science. The funder was not involved in the study design, collection, analysis, interpretation of data, the writing of this article, or the decision to submit it for publication.

### Acknowledgments

The authors thank Barbara Borlotti Amico (Nestlè Health Science) and Iside Morabito (Isi Events) for support, Mariateresa Cirillo for graphic design. The authors affiliated to the IRCCS Istituto Tumori "Giovanni Paolo II" in Bari are responsible for the views expressed in this article, which do not necessarily represent the Institute.

### Conflict of interest

The authors declare that the research was conducted in the absence of any commercial or financial relationships that could be construed as a potential conflict of interest.

### Publisher's note

All claims expressed in this article are solely those of the authors and do not necessarily represent those of their affiliated organizations, or those of the publisher, the editors and the reviewers. Any product that may be evaluated in this article, or claim that may be made by its manufacturer, is not guaranteed or endorsed by the publisher.
